# 997. RLS-0071 Improves Survival in a Rat Model of Intestinal Necrosis

**DOI:** 10.1093/ofid/ofab466.1191

**Published:** 2021-12-04

**Authors:** Kenji Cunnion, Parvathi Kumar, Brittany Lassiter, Katherine LaValle, Neel Krishna

**Affiliations:** 1 ReAlta Life Sciences, Norfolk, Virginia; 2 ReAlta Life sciences, Norfolk, Virginia

## Abstract

**Background:**

Intestinal necrosis and perforation are potentially life-threatening medical conditions that can lead to bacterial sepsis and systemic inflammatory response syndrome. Various etiologies can compromise the intestinal wall causing leakage of luminal contents including enteric bacteria and precipitate aggressive immunological responses including the complement system and neutrophils. RLS-0071 is a peptide inhibitor of the classical and lectin pathways and known modulator of various neutrophil mediated effectors including myeloperoxidase activity and NETosis.

**Methods:**

In this study we evaluated the extent to which immunomodulation via inhibition of the complement system and neutrophil effectors would alter survival in the setting of intestinal necrosis. Adolescent male Long-Evans rats were subject to cecal ligation and puncture (CLP) with one cohort receiving 40 mg/kg of RLS-0071 thirty minutes after surgery while the control group received no treatment. Survival of the rats was then assessed up to 5 days after surgery.

**Results:**

Animals treated with RLS-0071 demonstrated nearly 1.5-fold increase in survival compared to the untreated group. In order to further elucidate the increase in survival we explored inflammatory responses as assessed by markers of NETosis i.e., free DNA in plasma, and the pro-inflammatory cytokine, IL-6. A reduction in blood levels of free DNA and the inflammatory cytokine IL-6 were observed for animals treated with RLS-0071.

RLS-0071 increases survival of rats after cecal ligation

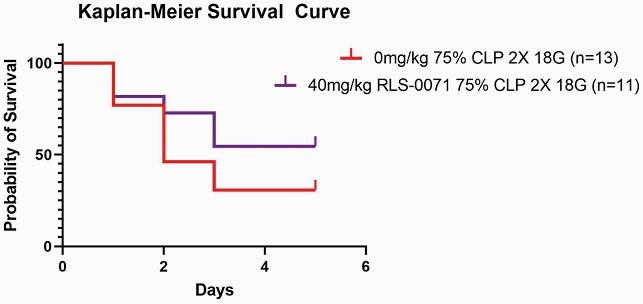

Kaplan-Meier survival curve assessment. The red line indicates the outcome after 75% CLP in animals not receiving treatment (n=13), whereas red curves represent the outcome after animals received a single dose of 40 mg/kg RLS-0071 (n=11).

RLS-0071 reduces free DNA levels in the blood

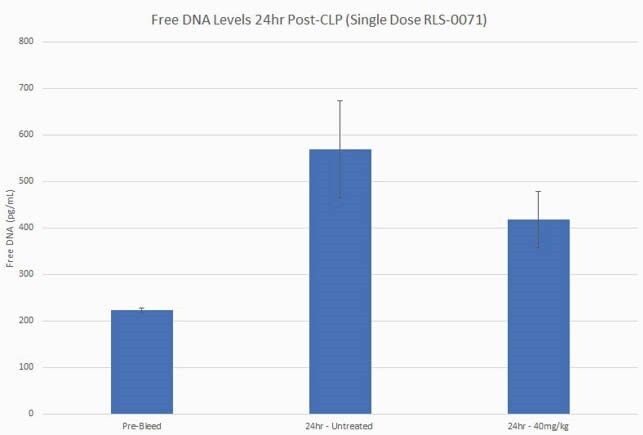

Plasma was isolated before surgery (pre-bleed) (n=7) and from animals subject to CLP with (n=4) and without (n=3) RLS-0071 administration 24 hours post-surgery. Plasma samples were incubated with PicoGreen. Fluorescence was read at an excitation wavelength of 485 nm and an emission wavelength of 520nm in a microplate reader. Data are means and standard error of the means

RLS-0071 reduces IL-6 levels in the blood

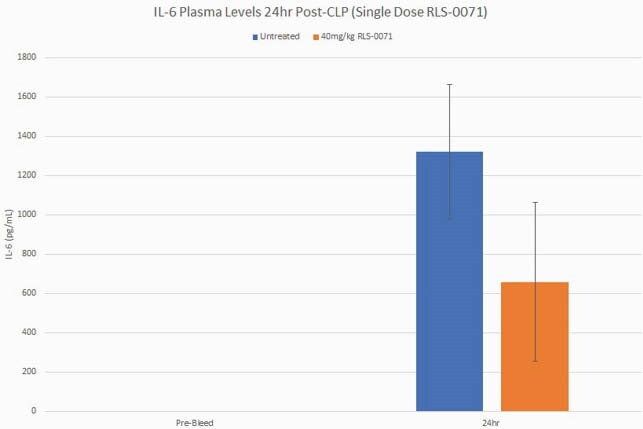

Plasma was isolated before surgery (pre-bleed) (n=8) and from animals subject to CLP with (n=3) and without (n=5) RLS-0071 administration 24 hours post-surgery. Plasma samples were analyzed in an IL-6 ELISA according to the manufacturer’s instructions. Data are means and standard error of the means.

**Conclusion:**

The results of these experiments demonstrate that RLS-0071 can increase survival after intestinal perforation by multi-pronged modulation of complement activation, neutrophil immune mechanisms and cytokine mediated inflammatory responses.

**Disclosures:**

**Kenji Cunnion, MD, MPH**, **ReAlta Life Sciences Inc** (Board Member, Employee, Shareholder) **Parvathi Kumar, MBBS**, **ReAlta Life Sciences Inc** (Employee) **Brittany Lassiter, BS**, **ReAlta Life Sciences Inc** (Employee) **Katherine LaValle, DVM**, **ReAlta Life Sciences Inc** (Employee) **Neel Krishna, PhD**, **ReAlta Life Sciences Inc** (Employee, Shareholder)

